# Using Facebook to Recruit Young Adult Veterans: Online Mental Health Research

**DOI:** 10.2196/resprot.3996

**Published:** 2015-06-01

**Authors:** Eric R Pedersen, Eric D Helmuth, Grant N Marshall, Terry L Schell, Marc PunKay, Jeremy Kurz

**Affiliations:** ^1^RANDDepartment of Behavioral and Policy SciencesSanta Monica, CAUnited States; ^2^Boston UniversitySchool of Public HealthBoston, MAUnited States

**Keywords:** alcohol, Facebook, Internet, mental health, online, young adult veterans

## Abstract

**Background:**

Veteran research has primarily been conducted with clinical samples and those already involved in health care systems, but much is to be learned about veterans in the community. Facebook is a novel yet largely unexplored avenue for recruiting veteran participants for epidemiological and clinical studies.

**Objective:**

In this study, we utilized Facebook to recruit a sample of young adult veterans for the first phase of an online alcohol intervention study. We describe the successful Facebook recruitment process, including data collection from over 1000 veteran participants in approximately 3 weeks, procedures to verify participation eligibility, and comparison of our sample with nationally available norms.

**Methods:**

Participants were young adult veterans aged 18-34 recruited through Facebook as part of a large study to document normative drinking behavior among a large community sample of veterans. Facebook ads were targeted toward young veterans to collect information on demographics and military characteristics, health behaviors, mental health, and health care utilization.

**Results:**

We obtained a sample of 1023 verified veteran participants over a period of 24 days for the advertising price of approximately US $7.05 per verified veteran participant. Our recruitment strategy yielded a sample similar to the US population of young adult veterans in most demographic areas except for race/ethnicity and previous branch of service, which when we weighted the sample on race/ethnicity and branch a sample better matched with the population data was obtained. The Facebook sample recruited veterans who were engaged in a variety of risky health behaviors such as binge drinking and marijuana use. One fourth of veterans had never since discharge been to an appointment for physical health care and about half had attended an appointment for service compensation review. Only half had attended any appointment for a mental health concern at any clinic or hospital. Despite more than half screening positive for current probable mental health disorders such as post-traumatic stress disorder, depression, anxiety, only about 1 in 3 received mental health care in the past year and only 1 in 50 received such care within the past month.

**Conclusions:**

This work expands on the work of other studies that have examined clinical samples of veterans only and suggests Facebook can be an adequate method of obtaining samples of veterans in need of care.

**Trial Registration:**

Clinicaltrials.gov NCT02187887; http://clinicaltrials.gov/ct2/show/NCT02187887 (Archived by WebCite at http://www.webcitation.org/6YiUKRsXY).

## Introduction

### Background

Young adult American veterans from the conflicts in Iraq and Afghanistan (Operation Enduring Freedom/Operation Iraqi Freedom, OEF/OIF) are at increased risk of mental health problems such as post-traumatic stress disorder (PTSD), anxiety, and depressive disorders, and/or substance use disorders [1-4]. Rates of these mental health problems are particularly concerning among young adult OEF/OIF veteran samples compared with active duty and civilian samples [2,5-8].

Although mental health problems evident among the growing group of veterans are a cause for concern, the majority of the research that is conducted with young veterans comes from samples recruited through the Veterans Health Care System (Department of *Veterans Affairs or VA*) or from examining VA administrative data [1,4,9]. Although this research is important to understand the needs of veterans in the VA, it excludes hundreds of thousands of veterans who do not seek VA care. Indeed, approximately 50% of OEF/OIF veterans do not seek services at the VA [10,11] and it is estimated that of the over 303,000 OEF/OIF service members and veterans with probable diagnoses of PTSD or depression, only half had sought help for any mental health problems from a medical or specialty provider [2]. Thus, while research on the health of veterans who are in the VA is critical for many reasons, it would be helpful to supplement those samples with research that recruits from other segments of the veteran population.

### Facebook Recruitment of Participants

Facebook, a social media website founded in 2004, is a promising—but largely unexplored—vehicle for reaching large numbers of OEF/OIF veterans for research and treatment outreach efforts. Facebook is the second most visited website in the United States [12], with over 165 million regular users from the United States alone [13]. More than half of Facebook users in the United States are under the age of 35 [14] and approximately two thirds to three quarters of all 18-34-year olds have a personal profile on the social media website such as Facebook [15,16].

Facebook allows users to connect with friends online through sharing personal updates or digital content such as pictures or Web pages. It allows users to endorse (by “liking” someone’s picture for example), discuss, or republish content posted by their friends and by organizations, commercial products and brands, media companies, news outlets, and more. Sharing and interacting with Facebook content personalizes a news feed on each user’s main Facebook page and drives an audience targeting engine for Facebook’s paid advertising products.

Compared with traditional forms of recruiting participants outside of a clinical setting (eg, posting flyers, newspaper advertisements), Facebook is well suited to reaching young adults for mental health research and is not biased toward one particular gender [17]. Facebook may also benefit longitudinal retention in research, which is often affected by inability to locate participants who have moved or changed contact information [18,19]. Ads on Facebook have been used successfully to recruit “hard-to-reach” populations such as sexual minorities, veterans interested in PTSD care, and other participants not accessed through traditional recruitment strategies [19-21], as well as young adults and adolescents for sensitive research areas such as substance use behavior [22-24], exposure to violence [25], mental health concerns [17,26], and sexual practices and women’s health issues [27-31]. Overall, Facebook is emerging as a viable and appropriate method of reaching populations for online surveys of a variety of physical and mental health issues [32].

Facebook- and general Internet-based research comes with both benefits and drawbacks [33-35]. For example, Internet-based recruitment is generally cheaper and faster than mailed surveys or interviews and can be used to access populations hesitant to participate in person. Internet surveys and programs enable participants to complete surveys at their convenience. However, lack of Internet and computer/mobile phone access still constitutes a barrier to participation for some classes of individuals in the population, and may effectively exclude the indigent or homeless. However, many of these same concerns hold true for traditional recruitment strategies as well (eg, phone-based interviews, television, and newspaper advertisements). Another potential problem is that, relative to in-person survey research (and to an extent, phone-based surveys), it is easier for respondents to misrepresent themselves; for example, participants misrepresenting eligibility to obtain an incentive (eg, payment, treatment) for which one is not eligible. As interest in Internet-based research (and more specifically the use of Facebook ads to recruit for Web-based research) has grown over the past 10 years, researchers have developed a series of procedures to deter misrepresentation of participants and best ensure validity of the sample obtained [36]. While these procedures help minimize concerns, more research is needed to better understand methods to reduce misrepresentation in Facebook-recruitment studies and ensure adequate representation of the populations targeted.

### Study Protocol

This study was the first phase of a larger clinical trial (National Institutes of Health NCT02187887) to provide young adult veteran drinkers with a personalized normative feedback intervention to reduce problematic alcohol consumption. For the first phase, we collected data on drinking behavior and attitude norms for use in the intervention phase of the study. An aim of this first phase of the study is also to examine the feasibility of recruiting a young adult veteran sample using Facebook. Nearly all young adult veterans report access to and use of the Internet, with the majority using the Internet daily and over two thirds reporting routine use for receiving health information or finding services [37-39]. In addition, their family members and friends are on Facebook, and thus, making connection with non-Facebook veterans may be a possibility through these referring sources. For example, Facebook groups tailored toward young adult veterans such as Iraq and Afghanistan Veterans of America have over 500,000 followers, with the most recent report from 2010 indicating that there are about 80,000 OEF/OIF veteran followers [40]. Young adult veterans are online and on Facebook, and thus, this paper describes Facebook recruitment of these individuals and provides findings on the cost and speed of using such a strategy to collect young veteran samples. Second, as Facebook is a promising yet novel method of reaching veterans for research, we aimed to look at the representativeness of our obtained sample. We compare demographic information from our sample with veteran population data from the American Community Survey (ACS) and information on the population of discharged military personnel available from the Department of Defense (DoD). Finally, we describe our sample, including health behaviors such as alcohol and marijuana use, mental health status, health care utilization, demographics, and military characteristics to provide a picture of what the young adult veteran sample from Facebook looks like in these areas.

## Methods

### Facebook Advertising

All procedures for advertising, consent, and survey methods were approved by the Institutional Review Board at the institution where the study was conducted. A series of Facebook ads targeted young adult veterans between the ages of 18 and 34 who had previously served in the US Air Force, Army, Marine Corps, or Navy. These ads targeted young adults likely to be veterans as well as Facebook users that might know a veteran who could be interested in our study. Ads were targeted to a potential audience of about 3.6 million Facebook users in the United States through a series of targeting criteria based on location (United States), age (“18-40”; however, we targeted beyond the 18-34-year-old age group in case a nonveteran family member/friend knew a young adult veteran), and interests (eg, “veteran,” names of national veteran service organizations such as Iraq and Afghanistan Veterans of American, movies and TV shows with an OEF/OIF focus such as *Restrepo* and *Generation Kill*, video games such as the *Call of* Duty series, “military spouse”). The study was named the Veterans Attitudes Online Survey Study and the Facebook ads did not specifically target any particular physical or mental health behaviors or problems.

**Figure 1:**
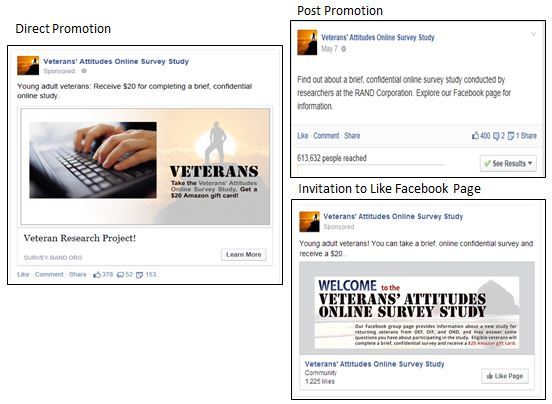
Examples of Facebook ads.

The following 3 types of ads were used: (1) direct promotion of the survey website, (2) promotion of posts we made to our Facebook page, and (3) invitations to “like” (publicly endorse) our Facebook page. Example ads are displayed in Figure 1. Direct survey website promotion ads were displayed on sidebar ad panels and in the personalized news feed that is the home page for Facebook users. These ads briefly described the study and allowed an individual to click through to the survey website. All direct promotion ads mentioned incentives for participation. Post-promotion ads were displayed in news feeds only, and included an option to reach our Facebook page, which contained information about our study and a link to the survey website. One of the 5 post-promotion ads discussed the incentive. Invitations to “like” our Facebook page were displayed in news feeds, with a suggestion that the reader might be interested in our page alongside a button to “like” our page directly from the ad. All of these ads also discussed the incentive. Both post promotions and invitations to like our Facebook page were aimed at cultivating ongoing interest and interactions with our study and to encourage social sharing of the survey info with friends. For all 3 sets of ads, Facebook users could “like” the ad, comment on the ad (eg, “This looks like a great study” would appear in the comments section under our ad), or share it with friends (eg, “Hey, check out this survey for veterans” would display on someone’s Facebook wall for their friends to see). All 3 types of ads automatically utilized the social networks inherent to Facebook. For example, when someone liked our ad or our Facebook page, this fact was promoted to that users’ friends in their news feed that “[Your friend] liked [our Facebook page] (or [our ad])”.

### Facebook Recruitment

The number of steps for a potential participant to access the survey from a Facebook promotion differed by the type of ad. If a Facebook user saw a direct survey website promotion ad, they could click on the ad itself and were directed to the survey Web page. The Web page contained a button that said “click here to access the survey” and contained 3 sentences describing that the study was conducted by researchers at RAND, responses were confidential, and contained a link to our Facebook page if the user wanted more information before clicking through. Alternately, if Facebook users saw a post promotion or invitation to like the page, they needed to click on that ad to first reach our Facebook page, where the link to the Web page was displayed.

If participants clicked through an ad directly to the survey Web page, it was anonymously recorded by Facebook as a “website click.” Participants reaching the Web page were presented with a consent form at the beginning the survey. If they consented, they accessed the survey. Participants were given a US $20 Amazon gift card for completing the survey. All user actions on our ads, including website clicks, likes, shares, and comments were anonymously recorded for aggregate reporting by Facebook. Facebook ad results reported below were generated from the detailed analytics reports provided by Facebook to all advertisers, for use in evaluating the effectiveness of paid ad campaigns. All reported findings are for unique user counts of each action that exclude any duplicate actions by a given Facebook account.

We followed procedures discussed by Kramer and colleagues [36] to reduce misrepresentation of participants and limit fraudulent responders. These procedures included prohibiting open access to the survey-hosted website, requiring screening questions to prevent and remove noneligible individuals from continuing to complete the survey, asking participants “insider knowledge” questions, examining time stamp of survey initiation and completion, identifying pairs of items that needed to be consistent, and verifying that individuals’ responses were consistent with previous research targeting veterans. We also restricted access to the survey website through a single login per Facebook account. That is, to access the survey, participants needed to login via their Facebook accounts, and we limited survey access to one completion per Facebook account. The information technology department at RAND worked with Facebook to ensure we were not collecting any information from a Facebook user’s profile (eg, list of friends) or personal information (eg, passwords) and that Facebook had no access to the data collected in our survey.

**Figure 2:**
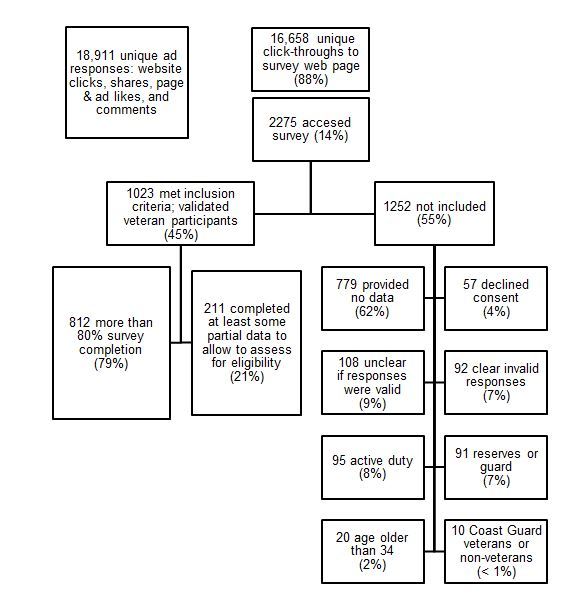
Flow diagram of sample participants.

Once individuals accessed the survey by clicking through to the Web page, they saw an informational statement describing eligibility, confidentiality, and other aspects of informed consent. If interested, they indicated agreement to participate in the study. They began the survey with screening questions of age, veteran status (eg, veteran, active duty, reserves/guard), and branch of service. To be eligible, veterans needed to be between 18 and 34 of age and fully separated from the military; thus, not currently in the reserves or guard units. We specified veterans of OEF/OIF in the recruitment documentation, but did not exclude veterans who were not involved in those combat operations. Eligibility criteria were made clear on our Facebook page and in consent. Respondents who were ineligible based on their responses to the screener were exited from the survey without ability to reenter. Figure 2 contains a description of the individuals who were screened out due to ineligibility. Next, participants were presented with questions about pay grade at discharge (eg, E-4); rank at discharge (eg, captain); and occupation code: military occupational specialty for Army and Marine Corps, enlisted classification (for Air Force), or specialty code (for Navy). These items were all open-ended responses. We used these 3 items, branch of service, and age to ensure consistency and verify participants had military knowledge consistent with military service. When it was unclear (eg, if veteran endorsed pay grade at discharge for both pay grade and rank items), we examined the rest of the individual’s data to determine whether their data appeared consistent with military service. We excluded participants in cases where data were still unclear or where misrepresentation was likely (Figure 2).

### Comparison and Description of the Sample

We sought to compare our sample with the national population of veterans. Data on the population were obtained from the ACS through the US Census Bureau’s online data extraction tool, DataFerrett. The 3-year estimates of population variables from the 2010 to 2012 ACS were selected. From this set of data we selected only cases from individuals aged 18-34 who had served in the active duty military in the past (but were not currently serving). In addition to age and military service, we also included gender, marital status, education level attained, income, and race/ethnicity in the data extraction. Because ACS data did not collect information on branch of service, we also examined reports prepared for the DoD [41] to determine the percentages of veterans from each of the 4 branches of service. From these published reports, we determined the average percentage of personnel separated from the US Armed Forces over the past 5 years (2008-2012) for Air Force, Army, Marine Corps, and Navy. Although we were unable to extract data to match veterans aged 18-34 only, we excluded nonmedically retired personnel, as the military requires 20 or more years of service before nonmedical retirement; thus, excluding retired personnel that would be older than 34. Of note, both the ACS and DoD data defined “veteran” as including those in the reserves or guard, whereas we excluded those individuals from our study. Thus, a precise match on demographic characteristics would not be expected.

### Measures

#### Overview

In addition to obtaining demographics to compare our sample with the ACS and DoD data, we sought to describe our sample in greater detail to provide a picture of the Facebook veteran population. Thus, we included measures of health behaviors, mental health, health care utilization, and other demographics and military characteristics.

#### Health Behaviors

We assessed any alcohol use in the past 30 days with a single item “During the past 30 days, how many days did you have at least 1 drink of any alcoholic beverage such as beer, wine, a malt beverage, or liquor?” Those that indicated at least 1 drinking day received a follow-up question for binge drinking behavior, “Considering all types of alcoholic beverages, how many times during the past 30 days did you have (*5 if male, 4 if female)* or more drinks on an occasion?” Drinking questions were preceded by a graphic depicting standard drinks. Lifetime marijuana use was assessed with a single yes/no question “In your lifetime, have you ever used cannabis (marijuana, pot, hash, hashish)?,” which was followed by a single item for past 6 months, “Have you used any cannabis (marijuana, pot, hash, hashish) over the past 6 months?” These items are standard single-item measures used in multiple studies of young adults and veterans to assess health behaviors.

#### Mental Health

PTSD was assessed with a screener for PTSD, the Primary Care PTSD Scale [42]. Reliability of the scale was adequate in our sample (alpha=.87). The Patient Health Questionnaire-2 item (PHQ-2) [43] was completed as a screener for depression, and the Generalized Anxiety Disorder-7 Item Scale (GAD-7) [44] screened for GAD. Both scales displayed adequate internal reliability (*r*=.80 for the PHQ-2; alpha=.96 for GAD-7). Lastly, we used a single item included in the *Behavioral Risk Factor Surveillance System* from the US Centers for Disease Control and Prevention to assess for self-reported diagnosis of traumatic brain injury (TBI), which defined TBI and asked participants if a doctor or other health professional ever told them they had suffered from one. These measures are established as valid and reliable when used with military samples in previous work [45-47].

#### Health Care Usage

Veterans were asked if, since discharge they had ever attended an appointment at a VA (including VA hospitals or VA community-based outpatient clinics), a vet center, or a non-VA/vet center clinic, hospital, or doctor’s office for any issue related to (1) physical health care, (2) mental health care, (3) alcohol use concerns, (4) other substance use concerns, (5) a review for service compensation (eg, to receive VA benefits from an injury incurred while on active duty), or for (6) any other reason. For mental health care, participants indicated whether they had attended a mental health care appointment in the past 12 months and if they had attended an appointment in the past 30 days. These items were modified from previous work assessing health care usage among service members and veterans [2].

#### Other Demographics and Military Characteristics

Participants indicated whether they were currently attending college and if so, what type of college they were attending (community, technical, state university, private college, or university) and whether they were using their GI Bill benefits or not. Participants were also asked how many children they currently had. They were asked how they heard about the study (ie, saw an ad on their computer, saw an ad on their phone, were forwarded a link to the survey from a friend/relative, a friend/relative told them where to find the survey link on Facebook) and on what device they completed the survey (eg, desktop/laptop, phone, tablet). Participants were asked how many times they had been deployed while on active duty. Lastly, they completed a yes/no measure of 11 deployment trauma experiences used in a previous work [2] to determine whether they had experienced trauma while deployed and if so, the severity of that trauma exposure (sum of yes responses to the 11 experiences).

## Results

### Facebook Advertising

#### Overview

Overall, the recruitment period lasted 24 days and we funded the Facebook ads for 12 of those days. Unanticipated delays with the advertising account payments, and an unexpected 4-day outage in the in-house server hosting our survey resulted in a total of 7 days during which ads were not actively shown. In addition, we stopped funding the ads 5 days before we took the survey down from our server. During these times, ads were not shown to participants on their Facebook news feeds, but they were still able to access our Facebook page and the see ads their friends shared or commented on from when the ads were running.

Over the recruitment period, 1.58 million Facebook users were shown an ad. On average, each person saw an ad about 2 times resulting in a total of about 3.3 million ad impressions. Of these, 18,911 unique individuals engaged with an ad (1.20%) by clicking through to our survey Web page or through liking, commenting on, or sharing the ad. A total of 16,658 users clicked through an ad to our Web page (1.05%) with the information statement and from there 2275 accessed the survey (0.14%). After a series of aforementioned checks, we obtained a total of 1023 verified veteran participants, representing 0.06% of the total targeted population (1023 of the 1,580,000 shown an ad; Figure 2). Overall, we spent US $7209 on ads for an overall cost per validated veteran participant of about US $7.05.

#### Direct Promotion

Direct promotion included ads that when clicked brought participants directly to the survey website. These ads utilized 72.01% (US $5191) of our budget and yielded 89.00% (16,831) of the 18,911 unique clicks. These cost about US $0.34 per unique click and yielded a 1.7% unique click-through rate.

#### Post Promotion

Post promotion included status posts on our page that did not have a direct link to the survey website. These utilized 25.00% (US $1802) of the budget and yielded 8.00% (1513) of the unique clicks. This type of ad produced a 0.7% unique click-through rate at a cost of about US $0.66 per unique click. The promotion was effective and reasonably cost effective for producing Facebook page likes, with 1094 likes at US $1.63 per like. Overall, this produced 1084 post likes, 104 post shares, 70 post comments, and 284 page likes, for a total of 1558 actions.

#### Invitations to like the Facebook Page

Invitation to like our Facebook page cost 3.00% (US $216) of the budget and yielded 3.00% (567) of the unique clicks. This method had the highest click-through rate at 3.00%, and cost about US $0.33 per unique click. This method produced 622 page likes at the lowest cost per like of all ad types.

### Facebook Recruitment

Figure 2 displays a flow diagram of the final sample, including reasons for exclusion of participants from the final sample. In total, we had 2275 individuals accessing the first page of the survey. Of these, 1023 (44.97%) were validated veteran participants who met our inclusion criteria and were verified through our validation procedures described earlier. A total of 812 participants completed more than 80% of the survey, whereas 211 completed at least enough partial data to allow for assessment of eligibility (ie, demographic questions at beginning of survey). The remaining 1252 (55.03%) individuals reached the survey but were not included in the sample due to incompletion and ineligibility. Most were not included because they provided no data after reaching the survey page—61.9% (776) of the remaining 1252 individuals. Of interest, 92 individuals endorsed responses that led us to believe they were not actually veterans or had illegitimate responses (eg, indicated impossible rank and pay grade combinations, weight under 50 pounds (22.6 kg), completed survey in less than 2 minutes) and 108 indicated responses that were unclear as to whether they were valid veteran participants.

We asked participants how they learned of our study. Of the 1023 validated veteran participants, 428 (41.8%) saw an ad on Facebook on the computer (eg, on the side bar of their Facebook news feed) and 379 (37.0%) saw an ad on the Facebook app on their mobile phone. A total of 102 (10.0%) reported that a friend/relative emailed them the link to the survey or directed them to the Facebook page where the survey link was hosted. Lastly, 114 (11.1%) indicated they learned about the survey after seeing a Facebook friend had “liked” one of our ads or our Facebook posts.

Participants reported on what device they completed the survey. As much as 21.9% of the participants (225/1023) did not complete this question. Of the remaining 798 who completed the item, 235 (29.4%) completed the survey on a personal desktop or laptop computer, 23 (3%) completed it on a public desktop or laptop computer (eg, a computer at the library), 451 (56.5%) completed it on their personal mobile phone, 72 (9%) completed it on a tablet (eg, an iPad or Samsung Galaxy), 8 (1%) completed the survey on someone else’s mobile phone, and less than 1% each completed it on someone else’s tablet (3 participants), on more than 1 device (ie, started the survey on tablet and finished on a laptop; 1 participant), or on a work computer (5 participants).

### Comparison and Description of the Sample

Participant demographics of the 1023 validated veteran participants with at least partial data are displayed in Table 1. As displayed in Table 1, participants were primarily male and white. About three quarters of the participants reported incomes under US $50,000, and about half were married. The sample consisted of veterans primarily from the Army and the Marines (84.9%, 869 participants). To determine how similar our obtained sample was to the general population of veterans, we compared our sample with the ACS data. As can be seen when comparing the first column of Table 1 (Facebook sample) with the ACS data column, our Facebook sample was similar to the broader ACS population on most demographic factors besides race/ethnicity, where our sample contained a higher percentage of Hispanic/Latino(a)s and fewer black/African Americans than might be expected in the general population of young adult veterans. In addition, when compared with the DoD population of separated military personnel, our sample contained substantially more Army and Marines than would be expected in the general separated population (43.1% and 19.8%, respectively), and fewer Air Force and Navy veterans than would be expected (15.3% and 21.8%, respectively). To account for these discrepancies when conducting subsequent analyses, we weighted our sample to match the population on branch of service (from the DoD data) and on race/ethnicity (from the ACS). Weighting on both branch and race/ethnicity appeared to best match our sample to the ACS (Table 1).

**Table 1:** Sample demographics of 1023 veteran participants with branch and ethnicity weights compared with American Community Survey and Department of Defense.

Variable		Facebook sample^a^ n/N (%)	Facebook sample weighted by branch and race/ethnicity n/N (%)	American Community Survey^b^ n/N (%)	Department of Defense^c^ n/N (%)
	Age (mean)	28.20 (SD 3.45)	28.24 (SD 3.63)	28.37 (SD 3.91)	
**Age (categories)**					
	<20	4/1023 (0.4)	20/1023 (2.0)	610/43,602 (1.4)	—
	20-24	155/1023 (15.2)	141/1023 (13.8)	7369/43,602 (16.9)	—
	25-29	485/1023 (47.5)	471/1023 (46.0)	16,482/43,602 (37.8)	—
	30-34	377/1023 (36.9)	391/1023 (38.2)	19,141/43,602 (43.9)	—
**Sex**					
	Male	905/1023 (88.5)	880/1023 (86.0)	35,143/43,602 (80.6)	—
**Race/ethnicity**					
	White	723/1023 (70.6)	697/1023 (68.1)	29,867/43,602 (68.5)	—
	Black or African American	37/1023 (3.6)	145/1023 (14.2)	5363/43,602 (12.3)	—
	Other	76/1023 (7.4)	71/1023 (6.9)	3009/43,602 (6.9)	—
	Hispanic/Latino(a)	188/1023 (18.4)	110/1023 (10.8)	5363/43,602 (12.3)	—
**Branch**					
	Army	616/1023 (60.2)	429/1023 (41.9)	—	334,591/776,313 (43.1)
	Marines	253/1023 (24.7)	193/1023 (18.9)	—	153,710/776,313 (19.8)
	Navy	87/1023 (8.5)	254/1023 (24.8)	—	169,236/776,313 (21.8)
	Air Force	68/1023 (6.6)	147/1023 (14.4)	—	118,776/776,313 (15.3)
**Marital status**					
	Married	527/1023 (51.5)	498/1023 (48.7)	20,667/43,602 (47.4)	—
	Divorced	177/1023 (17.3)	167/1023 (16.3)	4883/43,602 (11.2)	—
	Widowed	2/1023 (0.2)	1/1023 (0.1)	87/43,602 (0.2)	—
	Separated	54/1023 (5.3)	48/1023 (4.7)	1439/43,602 (3.3)	—
	Never married	247/1023 (24.1)	292/1023 (28.5)	16,525/43,602 (37.9)	—
	Other/member of unmarried couple	16/1023 (1.6)	17/1023 (1.7)	Not available	—
**Education**					
	Less than grade 12 or general educational development completion	33/1023 (3.2)	15/1023 (1.5)	567/43,602 (1.3)	—
	Grade 12 or general educational development (high-school graduate)	228/1023 (22.3)	207/1023 (20.2)	12,078/43,602 (27.7)	—
	Some college or technical school	610/1023 (59.6)	608/1023 (59.4)	23,414/43,602 (53.7)	—
	College 4 years or more (college graduate)	152/1023 (14.9)	193/1023 (18.9)	7543/43,602 (17.3)	—
**Income**					
	Less than US $10,000 to US $14,999	183/1023 (17.9)	204/1023 (19.9)	13,037/43,602 (29.9)	—
	US $15,000 to US $24,999	219/1023 (21.4)	205/1023 (20.0)	7194/43,602 (16.5)	—
	US $25,000 to US $49,999	357/1023 (34.9)	335/1023 (32.7)	14,040/43,602 (32.2)	—
	US $50,000 or more	264/1023 (25.8)	280/1023 (27.4)	933/43,602 (21.4)	—

^a^Defined veteran as discharged from Army, Marines, Navy, and Air Force. No reserves/guard. Data collected from April 2014.

^b^American Community Survey data from 3-year estimates (2010-2012) of those aged 18-34 only. Defined veteran as follows: "Has this person ever served on active duty in the US Armed Forces, Reserves, or National Guard?" We included those who indicated "Yes, on active duty during the last 12 months, but not now" and "Yes, on active duty in the past, but not during the last 12 months." This sample could include reserves/guard.

^c^Department of Defense data from average of past 5 years separated (2008-2012) excluding those who retired for nondisability reasons (N=776,313 separated between 2008 and 2012). This population could include reserves/guard.

To further describe our sample, we computed means and frequencies of health behaviors, mental health status, health care utilization, and other demographic factors on the unweighted sample and the sample weighted by branch and race/ethnicity. Both the unweighted and weighted samples were similar in their reports of these factors. As can be seen in Table 2, the majority of our sample drank alcohol in the past month and used marijuana within their lifetime, with over half of the drinkers reporting past month binge drinking and nearly half of lifetime marijuana users reporting use in the past 6 months. In addition, the sample appeared to be struggling with mental health concerns, with between one fifth and one half reporting a previous mental health diagnosis of TBI or screening positive for generalized anxiety, depression, or PTSD. About half of the sample reported any use of VA and non-VA services for mental health care and review for service compensation, with about three quarters receiving physical health care since discharge and about 15% (range 118-128) reporting receipt of alcohol or substance use care. As can be seen in Table 3, approximately two fifths were currently attending college and the majority of these students reported use of the GI Bill. Most veterans had at least 1 child. Finally, as might be expected from recruitment of an OEF/OIF sample of veterans, most reported some combat experience and reported a mean of about 2 deployments each.

**Table 2:** Health behaviors, mental health status, and health care utilization of the unweighted Facebook sample and the Facebook sample weighted by branch and race/ethnicity.

		Unweighted sample n/N (%)^a^	Sample weighted by branch and race/ethnicity n/N (%)^a^
**Health behaviors**			
	Alcohol use past 30 days	788/1023 (77.0)	788/1023 (77.0)
	Binge drinking^b^ past 30 days (*drinkers only*)	536/788 (68.0)	504/788 (64.0)
	Lifetime marijuana use	582/987 (59.0)	563/987 (57.0)
	Marijuana use past 6 months (*lifetime users only*)	279/582 (47.9)	239/582 (41.1)
**Mental health status**			
	Post-traumatic stress disorder^c^	385/819 (47.0)	401/819 (49.0)
	Depression^d^	360/819 (44.0)	311/819 (38.0)
	Generalized anxiety^e^	410/820 (50.0)	369/820 (45.0)
	Has a doctor told you that you have traumatic brain injury	221/820 (27.0) indicated “Yes”; 41/820 (5.0) indicated “do not know”	172/820 (21.0) indicated Yes; 49/820 (6.0) indicated do not know
**Health care utilization since discharge** ^f^			
	Physical health care	633/844 (75.0)	616/844 (73.0)
	Mental health care	490/844 (58.1)	439/844 (52.0)
	Past 12 months mental health care	354/844 (41.9)^g^	287/844 (34.0)^g^
	Past 30 days mental health care	17/844 (2.0)^h^	17/844 (2.0)^h^
	Alcohol use care	211/844 (25.0)	118/844 (14.0)
	Substance use care	203/844 (24.1)	127/844 (15.0)
	Review for service compensation	464/844 (55.0)	422/844 (50.0)
	Other (eg, marriage counseling)	203/844 (24.1)	143/844 (16.9)

^a^Denominators in n/N represent the number of participants who completed the item.

^b^Binge drinkers classified as 4 drinks for women, 5 drinks for men at any one time in the past 30 days.

^c^Primary Care Post-Traumatic Stress Disorder Scale score of 3 or higher indicates optimal screener for post-traumatic stress disorder diagnosis [42].

^d^Patient Health Questionnaire score of 2 or higher indicates optimal screener for depression diagnosis [43].

^e^Generalized Anxiety Disorder-7 Item Scale (GAD-7) score of 10 indicates moderate/severe symptoms of anxiety and optimal screener for generalized anxiety disorder diagnosis [44].

^f^Any use of Veterans Affairs Health Care System (VA), Vet Center, or non-VA since discharge.

^g^Percentage reflects entire sample. Of those who reported any mental health care use since discharge (490 unweighted; 439 weighted), 75.9% (372) of the unweighted sample and 69.9% (307) of the weighted sample reported past 12-month usage of mental health care.

^h^Percentage reflects entire sample. Of those who reported any mental health care use since discharge (490 unweighted, 439 weighted), 4.1% (20) of the unweighted sample and 5.0% (22) of the weighted sample reported past 30-day usage of mental health care.

**Table 3:** Other demographics including whether the participants are current students, number of children, military characteristics, and combat severity status of the unweighted Facebook sample and the Facebook sample weighted by branch and race/ethnicity.

		Unweighted sample n/N (%)^a^	Sample weighted by branch and race/ethnicity n/N (%)^a^
**Current student**			
	Not currently attending college	542/1023 (53.0)	593/1023 (58.0)
	Attending community college	133/1023 (13.0)	123/1023 (12.0)
	Attending a technical college	123/1023 (12.0)	61/1023 (6.0)
	Attending a state university	133/1023 (13.0)	133/1023 (13.0)
	Attending a private college or university	92/1023 (9.0)	113/1023 (11.0)
	Use of GI Bill (those attending college only)	352/434 (81.1)	369/434 (85.0)
**Children**			
	No children	368/1023 (36.0)	390/1023 (38.1)
	1 child	206/1023 (20.1)	235/1023 (23.0)
	2 children	204/1023 (19.9)	215/1023 (21.0)
	3 or more children	246/1023 (24.0)	183/1023 (17.9)
**Military characteristics**			
	Number of deployments	1.71 (SD 1.53) range 0-14	1.90 (SD 2.07) range 0-14
**Combat trauma**			
	Combat trauma experiences (any)	795/883 (90.0)	751/883 (85.1)
	Between 1 and 5 combat trauma experiences	453/795 (57.0)	390/751 (51.9)
	Between 6 and 11 combat trauma experiences	342/795 (43.0)	361/751 (48.1)

^a^Denominators in n/N represent the number of participants who completed the item.

## Discussion

### Principal Findings

This paper describes the methods used to recruit a sample of young adult veterans for a research study using the social media website Facebook. We sought to examine the feasibility of recruiting young veterans via this mechanism by documenting the process of recruitment, describing the sample on a number of demographic and health factors, and comparing the obtained sample with young adult veteran population-level data from national samples available from the ACS and the DoD. In sum, the recruitment period lasted approximately 1 month and yielded a sample of 1023 verified veteran participants for the advertising price of approximately US $7.00 per participant.

### Comparison and Description of the Sample

Compared with the ACS population-level data for young adult veterans, we recruited fewer African American/black veteran participants than we would expect given the young adult veteran population. Yet, we recruited a higher percentage of Hispanic/Latino(a)s than would be expected. Regarding branch differences compared with data from the DoD over the past 5 years, we recruited more Army and Marine veterans and fewer Navy and Air Force veteran than would be expected in the general population of separated military personnel. Other work using Facebook to recruit OEF/OIF veterans has similarly reported underrepresentation of Navy and Air Force veterans and African American/black veterans [21]. One of the reasons for this may be the manner in which Facebook targets advertisements, which in our case displayed ads to those whose Facebook posts and interactions suggested veteran status, affiliation with the military, and other interests a veteran might have. It is possible that Army and Marine veterans are more visible with their veteran/military-focused content on Facebook (as are their family members) and were targeted more often by our ads. Observational research using Facebook could help indicate if this is the case, as well as to determine whether African Americans/blacks are disproportionately less likely to have veteran/military-focused content on their pages while Hispanic/Latino(a)s are more likely to display such content. It is also possible that these discrepancies are due to racial/ethnic differences inherent to the US users of Facebook—about 75% white, 11% African American/black, 9% Hispanic/Latino(a)—[14], though statistics for veteran Facebook users by race/ethnicity are unknown. Although other work has looked at nonveteran samples (ie, adolescent girls) and found recruitment of racial/ethnic groups comparable across Facebook and non-Facebook recruitment methods [19], future experiments are needed to compare racial/ethnic minority recruitment rates between traditional recruitment methods and Facebook.

Our sample did not appear to be unrepresentative with respect to most demographic characteristics such as gender, age, income level, education level, or marital status. However, we did recruit about 8% more males than expected given the young adult veteran population, which fits with previous Facebook veteran research [21] but not with recruitment of adolescents [17]. In addition, prior work with young women found that Facebook recruitment yielded a sample of women from higher socioeconomic groups compared with lower ones [30]. Yet, we found comparable reports of income in our sample with the general young adult veteran population, which indicates the recruitment method did not exclude those from lower socioeconomic groups. Although we did not assess housing status (eg, homeless) or access to Internet, we did find that about 4% (31) of the sample completed the survey on a public computer or someone else’s computer or phone. Thus, it is possible that this method can be used to capture those without computers or Internet access, but more rigorous research on this topic is warranted.

### Reaching Veterans in Need of Mental Health Services

Despite not specifically advertising to veterans in need of mental health services, our sample yielded an unusually high number of veterans struggling with a variety of mental health concerns such as depression, PTSD, anxiety, and TBI, as well as those engaging in risky health behaviors such as binge drinking and marijuana use. Despite these high rates, only half had attended any appointment for a mental health concern, with only about 1 in 3 receiving mental health care in the past year and only about 1 in 50 receiving such care within the past month. Our sample appeared to have higher rates of mental health problems than community samples of veterans and service members [2,48,49] and OEF/OIF VA veterans [10,50]. Depending on the intended focus of a research study, obtaining more individuals with mental health concerns than would be expected in the general population could be desirable. More specifically, while this would be a problem for a study designed to estimate the rates of disorder in the population, it is a virtue for studies designed to identify individuals who could benefit from Web-based delivery of care for behavioral health problems, such as our broader intervention study. In general, recruitment of individuals into treatment studies on mental health has traditionally been difficult, with barriers to enrollment related to inconvenient scheduling times, lack of transportation to research sites, and stigma related to discussing sensitive matters with an unknown interviewer in a face-to-face setting [51]. Studies targeting active duty service members and young adult veterans with mental health concerns such as PTSD or TBI have similarly struggled with recruitment [52,53]. Yet, another study using Facebook recruitment has also been successful at obtaining OEF/OIF veteran participants in need of help for PTSD and hazardous alcohol use for an online intervention study [21]. Another study using Facebook has also indicated that those with mental health concerns may be more likely to complete surveys online than through the postal mail [17]. Combined with our findings, it is apparent that young veterans with mental health concerns are on Facebook and are willing to participate in research studies. This represents an important avenue in which to reach and provide outreach to those in need; both those seeking care and those not actively looking for help.

### Validation of the Survey Respondents

Lastly, there is a concern that Internet and Facebook studies may attract individuals misrepresenting themselves to receive incentives [36,54]. We included several verification checks to ensure to the best of our ability that we were capturing the young adult veterans we intended to recruit. Our within-survey procedures (eg, screening out those still on active duty or over the age of 34) and validation checks after data collection (eg, checking for consistent data) removed about 20.5% (257) of those who accessed our survey overall. It is unknown why over one third of individuals accessed the survey but decided to not pursue past consent. It is possible at the point of consent they realized the legitimacy of the study and chose to not misrepresent themselves at this point. It is also possible we lost actual potential veteran participants at this point, but we do not have the data to draw meaningful conclusions here.

In this study, 20.6% (211) of the verified veteran sample terminated the survey after initial demographic and health behavior questions. This is somewhat perplexing given the ease of online survey completion. Indeed, in the Millennium Cohort Study, Web-based recruitment was better than traditional paper invitations and surveys at yielding completed surveys from males and younger active duty personnel [55]. Although we do not have these data, we suspect that this rate of partial completion and low survey consent overall may be a product of the survey not being optimized for use on mobile phones. Indeed, over one third of our total sample learned about the study through mobile phone-based ads, about two thirds who completed the survey did so on their phone, and the majority of clicks to the survey website came from ads displayed on phones. While our survey converted adequately from the designed Web version (eg, 1 question per page, large font), it was not optimized for mobile viewing. Thus, it would likely take a participant a longer amount of time to fill out the survey on a phone and thus may explain drop off toward the latter portions of the survey. Online survey research may need to consider mobile phone-adapted surveys that are easy to access on mobile devices in a single sitting.

### Areas for Further Research and Recommendations

More research is needed to determine the cost effectiveness of Facebook for recruiting participants in more diverse veteran samples and across different populations. Although some studies have compared costs of different recruitment methods in biomedical and mental health studies [56-58], most journal articles do not include discussions of recruitment costs, and thus, comparisons between recruitment methods for specific targeted groups are difficult. Costs of any recruitment strategy will likely vary greatly depending on the targeted population; for example, recruitment of participants through Facebook has ranged from no cost for adolescent girls [19], about US $4 for young adults [22], about US $11 for pregnant women [31], about US $20 for depressed adults, and up to US $30 for veterans [59]. Comparisons of Facebook and other online advertisements with postal mailing recruitment strategies suggest that Facebook (at US $1.50 per completed survey) was more cost-effective than postal mailings (at about US $19 per completed survey) for recruiting those with mental health concerns [17]. Yet, demographics differed between samples (eg, younger people were more likely to be recruited online than by mail), which can have implications depending on the purpose of the study. To our knowledge though, there have been no published comparisons between Facebook recruitment strategies and traditional strategies (eg, mailings, TV, and radio ads) for the veteran population, which is an area for important future research work.

In this study, our sample was relatively inexpensive to recruit (about US $7 per validated participant plus a US $20 gift card incentive) and data collection for the single brief survey was completed rather quickly. Yet, this survey was designed to be completed in a single sitting. Studies requiring more commitment on the part of participants may or may not see similar success. For example, although Brief and colleagues [21] recruited 600 participants in 46 days using Facebook (with a cost of about US $30 per enrolled participant plus US $20 incentive for baseline assessment), attrition at the 2-month follow-up assessment was high (ie, 51.7% of the intervention group, 209/404, and 38.7% of the delayed intervention group, 76/196). Similarly, only about one third of participants completed all 8 modules of the intervention (ie, 33.9% of the intervention group, 137/404, and 38.7% of the delayed intervention group, 76/196). Thus, Facebook recruitment, although established as a viable method of recruiting veterans into research studies, should be tested further to examine how it can be used to retain participants in longitudinal work and if those who sign up for studies via Facebook (vs other recruitment strategies) are more or less likely to drop out of longitudinal studies that expand beyond a one-time brief survey.

We also recommend that studies recruiting from Facebook take steps to validate that participants meeting study eligibility criteria are not misrepresenting themselves (see Kramer and colleagues for guidance [36]). We also recommend researchers compare their Facebook-recruited samples with the best available population-level data to determine representativeness. While most convenience samples are limited in generalizability due to their nature, “Methods” section in journal articles could include information to allow for determination of the extent to which Facebook-recruited samples differ from relevant populations. Weights could be applied if necessary. Lastly, we recommend more research learning how to use other online social media sites to target veterans and other at-risk groups for inclusion in mental health survey and intervention studies. Widely used sites such as YouTube, Twitter, and LinkedIn have options for targeted advertising, as do sites that are focused on specific groups that may be of interest (eg, advertising research studies to men who have sex with men via the Grindr app).

### Limitations

There are additional limitations worth noting. First, by design to limit misrepresentation, participants needed a Facebook account, which excludes those who may have had Internet access but not a Facebook account. In addition, we recruited approximately 0.06% (1023/1,580,000) of the targeted Facebook population. Although this seems low, it should be noted that the targeted population included friends/family members of veterans, as well as others who did not have any veteran contacts to refer to the study (eg, someone who “liked” the *Call of Duty* video game but had no connection with US veterans). Still, the majority of users who clicked on our ads but did not click through to access the survey (ie., only 2275 of the 16,658 who clicked on an ad went on to take the survey) suggests a discrepancy between clicks and enrollment for which we do not have data to explain.

### Conclusion

The Internet is becoming an increasingly popular venue for reaching young people to deliver informational programs, stand-alone interventions, and adjunct treatments for a variety of mental health problems such as depression and heavy alcohol use [60-63]. This study suggests that the use of Facebook-based recruitment appears to be an inexpensive and practical method to reach young adult veterans for research studies. Understanding how to reach young veterans through Internet-based recruitment can help inform intervention/prevention programs and outreach efforts with this at-risk population. It has applicability to be a means to provide young veterans with resources and information about care seeking, as well as to provide stand-alone or adjunct treatments for mental health concerns. Internet programs and research studies have the ability to reach a widespread audience, can be less expensive than more intensive programs, require less staffing and expertise, can be conveniently accessible at all hours by consumers and, most importantly, can provide outreach and services for individuals who may have never otherwise engaged in such care.

## Abbreviations

ACS: American Community Survey
GAD: generalized anxiety disorder
GAD-7: Generalized Anxiety Disorder-7 Item Scale
OEF: Operation Enduring Freedom
OIF: Operation Iraqi Freedom
PHQ-2: Patient Health Questionnaire-2
PTSD: post-traumatic stress disorder
TBI: traumatic brain injury
VA: Department of Veterans Affairs
